# Pigmentary retinopathy, rod–cone dysfunction and sensorineural deafness associated with a rare mitochondrial tRNA^Lys^ (m.8340G>A) gene variant

**DOI:** 10.1136/bjophthalmol-2017-310370

**Published:** 2017-07-20

**Authors:** Jaidip S Gill, Steven A Hardy, Emma L Blakely, Sila Hopton, Andrea H Nemeth, Carl Fratter, Joanna Poulton, Robert W Taylor, Susan M Downes

**Affiliations:** 1 John Radcliffe Hospital, Oxford, UK; 2 Wellcome Trust Centre for Mitochondrial Research, Institute of Neuroscience, Newcastle University, Newcastle upon Tyne, UK; 3 Department of Clinical Genetics, Churchill Hospital, Oxford, UK; 4 Nuffield Department of Clinical Neuroscience, John Radcliffe Hospital, Oxford, UK; 5 Oxford Medical Genetics Laboratory, Churchill Hospital, Oxford, UK

**Keywords:** Mitochondrial DNA, mitochondrial DNA mutations, *MTTK*gene, mitochondrial eye disease

## Abstract

**Background/Aim:**

The rare mitochondrial DNA (mtDNA) variant m.8340G>A has been previously reported in the literature in a single, sporadic case of mitochondrial myopathy. In this report, we aim to investigate the case of a 39-year-old male patient with sensorineural deafness who presented to the eye clinic with nyctalopia, retinal pigmentary changes and bilateral cortical cataracts.

**Methods:**

The patient was examined clinically and investigated with autofluorescence, full-field electroretinography, electro-oculogram and dark adaptometry. Sequencing of the mitochondrial genome in blood and muscle tissue was followed by histochemical and biochemical analyses together with single fibre studies of a muscle biopsy to confirm a mitochondrial aetiology.

**Results:**

Electrophysiology, colour testing and dark adaptometry showed significant photoreceptor dysfunction with macular involvement. Sequencing the complete mitochondrial genome revealed a rare mitochondrial tRNA^Lys^ (*MTTK*) gene variant—m.8340G>A—which was heteroplasmic in blood (11%) and skeletal muscle (65%) and cosegregated with cytochrome *c* oxidase-deficient fibres in single-fibre studies.

**Conclusion:**

We confirm the pathogenicity of the rare mitochondrial m.8340G>A variant the basis of single-fibre segregation studies and its association with an expanded clinical phenotype. Our case expands the phenotypic spectrum of diseases associated with mitochondrial tRNA point mutations, highlighting the importance of considering a mitochondrial diagnosis in similar cases presenting to the eye clinic and the importance of further genetic testing if standard mutational analysis does not yield a result.

## Introduction

Mitochondrial DNA (mtDNA) disease can be caused by mutations (single-nucleotide substitutions or large-scale rearrangements) that arise either in the mitochondrial genome (primary mtDNA disorders) or in nuclear genes whose protein products are intimately linked to mtDNA maintenance, replication or repair.^1^ According to recent estimates, mtDNA mutations causing clinically manifesting disease affect 1 in 5000 individuals.^2^ To date, as many as 300 distinct, disease-causing mutations within the mitochondrial genome have been characterised, and of these, the vast majority involve the genes coding the 22 mitochondrial transfer RNA (mt-tRNA) molecules.^3^ Furthermore, just three genes—*MTTL1, MTTI* and *MTTK* encoding mt-tRNA^Leu(UUR)^, mt-tRNA^Ile^ and mt-tRNA^Lys^, respectively—harbour over a third of all mt-tRNA mutations. Point mutations in mt-tRNA genes are thought to impair overall mitochondrial translation as a result of alterations to mt-tRNA secondary structure.^1^

Mt-tRNA mutations have been reported to cause a wide range of clinical disorders with considerable phenotypic heterogeneity, variable age of onset and disease severity.^4^ These differences, aside from presenting a diagnostic challenge, make the prevalence of pathogenic mitochondrial mutations difficult to ascertain. One of the factors contributing to the variable penetrance observed in many mtDNA-associated diseases is the phenomenon of heteroplasmy due to a multicopy mitochondrial genome. In patients with pathogenic mtDNA mutations, each cell contains both mutated and wild-type mitochondrial genomes, and as such the proportion (or ‘ratio’) of mutated to wild-type genomes in a given tissue contributes markedly to the age at onset, severity and extent of the phenotype. In most cases, mutation load within a specific tissue has to exceed a critical threshold level of mtDNA mutation level for manifest disease to be present, which can also contribute to a delay in making a diagnosis.^1^

Mt-DNA diseases can be broadly classified into multisystemic syndromes including maternally Inherited diabetes and deafness (MIDD), neurogenic weakness, ataxia and retinitis pigmentosa, myoclonic epilepsy and ragged-red fibres (MERRF) and mitochondrial encephalomyopathy, lactic acidosis and stroke-like episodes or isolated, organ-specific diseases such as myopathy (including ocular myopathy), sensorineural deafness, retinopathy or cardiomyopathy.^5^ Ophthalmic manifestations are commonly seen in mitochondrial disorders and include bilateral optic neuropathy, progressive external ophthalmoplegia and ptosis, pigmentary retinopathy and retro-chiasmal visual loss.^6^

More specifically, pathogenic mutations in the *MTTK* gene predominantly result in multisystemic disorders that affect the central nervous system including the MERRF syndrome.^7^ Jeppesen and colleagues recently described a patient with a heteroplasmic m.8340G>A *MTTK* variant manifesting as isolated myopathy.^8^ Here, we report a case of a 39-year-old man harbouring the identical mtDNA point mutation with an extended phenotype of childhood epilepsy, migraines and sensorineural deafness who presented to eye clinic with night blindness and cataracts. Ophthalmic assessment revealed pigmentary retinopathy with rod and cone cell dysfunction.

## Patient and methods

### Case report

The patient was born to non-consanguineous Caucasian parents following an uncomplicated pregnancy with a normal delivery at term, but had episodes of febrile convulsions as a toddler. His development was otherwise normal with no significant psychomotor delay or learning difficulties. At 5 years of age he was diagnosed and treated for myoclonic epilepsy. He stopped treatment at the age of 15 years and has remained seizure free since. As a child, the patient was noted to be of short stature and was diagnosed with growth hormone deficiency for which he received growth hormone supplementation. His medical history was also relevant for recurrent migraines starting as a teenager, but with a decrease in severity and frequency over the last 20 years. From the age of 20, he developed sensorineural hearing loss necessitating the use of hearing aids—a condition he had initially attributed to working as a drumming instructor. Family history of note includes: short stature (5 ft 1 in) in both parents and deafness in a maternal great-grandfather.

By 31 years, he had developed bilateral night blindness and photosensitivity with glare. At that time, he had not noted any gait disturbance or muscle weakness.

### Clinical findings

Clinical examination revealed short stature with normal muscle bulk. However, he had elbow contractures and a stiff spine. Power was normal in all muscle groups except mildly reduced (Medical Research Council grade 4+/5) in ankle eversion. Reflexes were brisk with no evidence of any ataxia.

**Figure 1:**
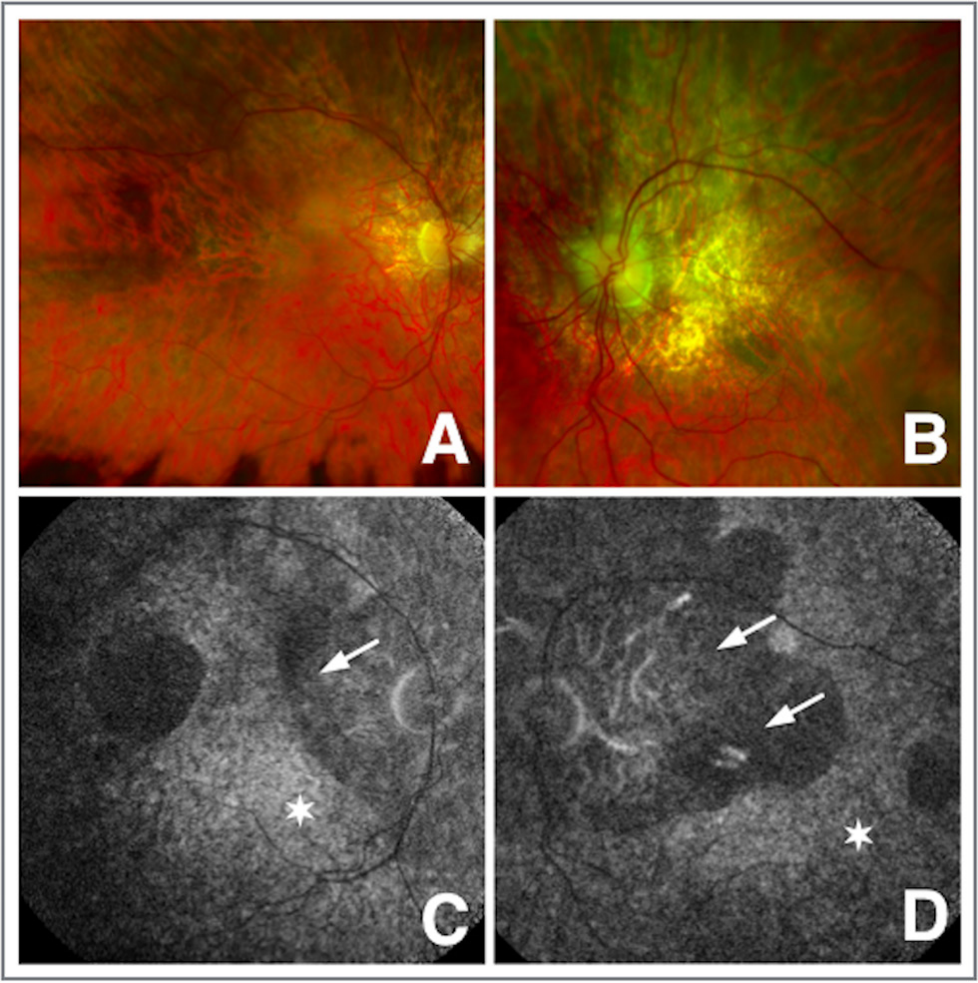
Colour fundus photographs and fundus autofluorescence (AF). (A and B) Colour fundus photographs (Optos PLC, Dunfermline, Scotland, UK) showing bilateral posterior pole atrophic changes more pronounced in the left eye. (C and D) Autofluorescence showing grossly reduced signal in the peripapillary region in both eyes (arrows), encroaching on the central macula in the right eye with a temporal area of AF loss in the left eye. Both show areas of increased stippled autofluorescence signal representing disturbed affected retinal pigment epithelium (stars).

**Figure 2:**
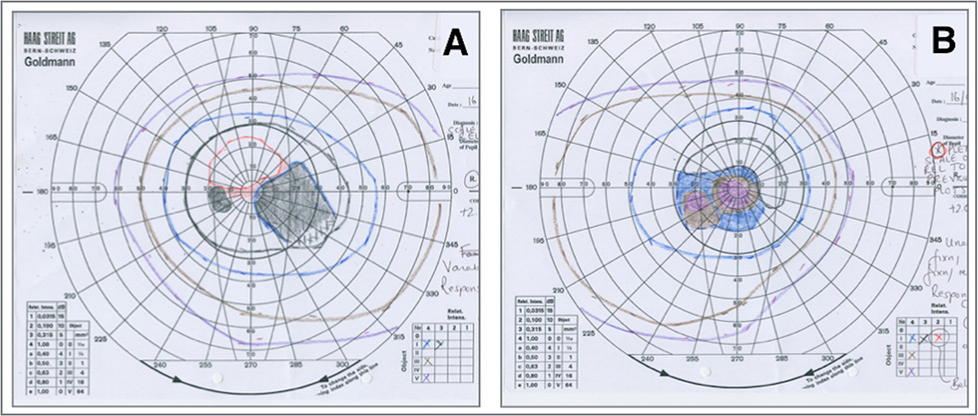
Goldmann visual fields showing paracentral scotomas in the right (A) field and centrocaecal scotoma in the left (B).

Ocular examination revealed visual acuities of Right: 6/18 and Left: 6/9 unaided. There was no evidence of ptosis or ophthalmoplegia. Intraocular pressure measurements were normal. Early cataracts were noted at first presentation, which progressed, requiring cataract surgery. Funduscopy revealed granular, pigmentary mottling affecting the macula accompanied by atrophic patches, with punctate light and dark pigmentation consistent with a ‘salt-and-pepper’ retinopathy. There was mild vascular attenuation but no intraretinal bone spicule pigmentation (figure 1A, B). Autofluorescence (Heidelberg Retina Angiograph, Heidelberg Engineering, Heidelberg, Germany) revealed a grossly reduced signal particularly affecting the maculae consistent with atrophy (figure 1C, D). Goldmann kinetic perimetry showed paracentral scotomas in the right visual field and centrocaecal scotoma in the left (figure 2). Dark adaptometry showed minimal cone adaptation and no dark adaptation after 45 min. Colour contrast sensitivities revealed elevated tritan and protan thresholds in both eyes. Full-field electroretinography (ERG) revealed reduced scotopic and photopic amplitudes and increased implicit times demonstrating involvement of both photoreceptor systems, which showed similar results when repeated a year later. Electro-oculography (EOG) showed a reduction in light rise (left 109%; right 128%; table 1).

**Table 1:** Table of electrophysiology details

Dark adaptometry	Cone	Rod
Final dark adapted thresholds after 45 min (log units)	−1.4	−1.0
EOG	OD	OS
Arden Index (%) [>200%]	109	128
Rod ERG	Rod ERG	
	OD	OS
a implicit time (ms) [38.86+/-3.31]	25	24
b implicit time (ms) [85+/-19.56]	59	58
a amplitude (μV) [52.29+/-51.21]	62	34
b amplitude (μV) [250.2+/-143.3]	57	62
Cone ERG		
	OD	OS
a implicit time (ms) [16.42+/-1.34]	18	17
b implicit time (ms) [27.75+/-1.73]	36	38
a amplitude (μV) [38.52+/-23.95]	11	5.0
b amplitude (μV) [128.7+/-46.9]	16	9.0
Maximal ERG (standard flash)		
	OD	OS
a implicit time (ms) [16.86+/-3.41]	25	24
b implicit time (ms) [46.79+/-14.74]	59	58
a amplitude (μV) [179.8+/-106.1]	62	34
b amplitude (μV) [333.2+/-201.1]	57	62
PERG P50	OD	OS
Implicit time (ms) [51.6+/-2.8]	NMC	59
Amplitude (μV) [4.1+/-1.7]	NMC	1.5
30 Hz ERG	OD	OS
Implicit time (ms) [24.33+/-1.97]	36	37
Amplitude (μV) [106.8+/-32.55]	21	19

*Normal values in square brackets.

EOG, electro-oculogram; ERG , electroretinogram; ms, milliseconds; NMC, no measurable components; OD, right ; OS, left; PERG, pattern electroretinogram; μV, microvolts.

## Results

### Mitochondrial genetic studies

A peripheral blood leucocyte sample was screened for common mtDNA point mutations (m.3243A>G, m.8344A>G, m.8993T>G/C) and primary Leber Hereditary Optic Neuropathy mutations, but no causal variants were identified. Subsequently, sequencing of the complete mitochondrial genome (Ion Torrent next-generation sequencing) was undertaken to screen for novel or rare pathogenic mtDNA variants, revealing the m.8340G>A *MTTK* change at a low level of heteroplasmy (11% in blood). A skeletal muscle biopsy was taken to confirm a diagnosis of mitochondrial disease, revealing the m.8340G>A variant at an intermediate level of heteroplasmy (65% as determined by quantitative pyrosequencing).

Quantitative pyrosequencing of DNA samples obtained from the patient’s mother showed no evidence of the m.8340G>A mutation in buccal epithelia, urinary sediment or a blood sample implying the pathogenic mutation had arisen de novo during embryogenesis and not been maternally inherited.

### Muscle histopathology, biochemistry and single-fibre segregation studies

**Figure 3:**
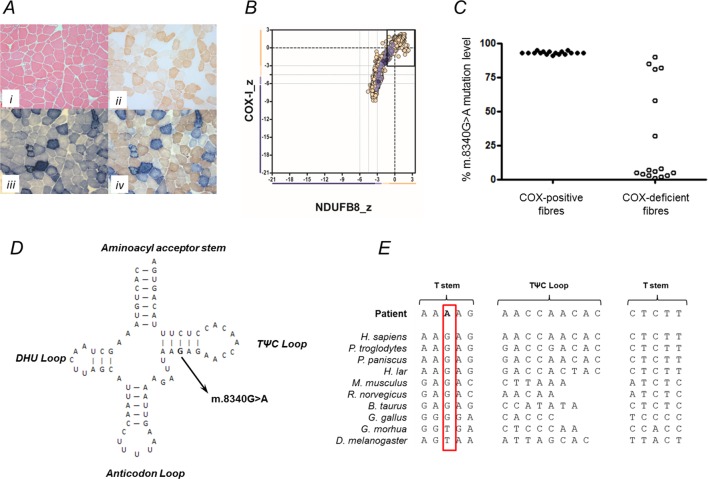
Mitochondrial histopathological, biochemical and molecular genetic findings associated with the m.8340G>A MTTK variant. (A) Muscle histology and histochemistry in our patient including H&E (i), COX (ii), SDH (iii) and COX-SDH (iv) reveal numerous COX-deficient, ragged-red fibres. (B) Quadruple immunofluorescence profile shows loss of both complex I (NDUFBA) and complex IV (COX-1) subunits, confirming a multiple mitochondrial respiratory chain disorder. (C) Single-fibre PCR analysis of the m.8340G>A variant demonstrating segregation with a biochemical defect in individual COX-deficient muscle fibres. (D) Schematic representation of the mt-RNA^Lys^ cloverleaf structure, illustrating the position of the m.8340G>A variant in the TψC stem. (E) Phylogenetic conservation of the appropriate region of the mt-tRNA^Lys^ gene sequence showing that the m.8340G>A variant affects an evolutionary conserved residue involved in Watson-Crick base pairing.

Muscle biopsy analysis showed remarkable mitochondrial histochemical abnormalities characterised by a mosaic pattern of cytochrome *c* oxidase (COX) deficiency affecting ~40% of all fibres on both the individual enzyme reaction and the sequential cytochrome c oxidase-succinate dehydrogenase (COX-SDH) reaction (figure 3A) and subsarcolemmal mitochondrial accumulation (ragged-blue fibres affecting ~5% of the total biopsy) on the individual SDH reaction (figure 3A), consistent with a mitochondrial aetiology. Quadruple oxidative phosphorylation (OXPHOS) immunofluorescence undertaken according to Rocha *et al* 9 confirmed the presence of muscle fibres lacking both complex I (NDUFB8) and complex IV (COX-1) expression, confirming a multiple respiratory chain defect (figure 3B). Single muscle fibre analysis of individual COX-positive and COX-deficient fibres revealed a statistically significant higher mutation load in COX-deficient fibres (93.12%±0.26% (n=17 fibres)) than in COX-positive fibres (COX-positive fibres: 29.50±8.97 (n=16 fibres); p<0.0001) confirming pathogenicity of the m.8340G>A variant (figure 3C).

Our patient was subsequently referred to a specialist mitochondrial clinic for follow-up and counselling with regular diabetic screening, cardiology review, ophthalmology review and lung function tests to monitor his condition.

## Discussion

We describe a patient with a rare mtDNA variant associated with primarily an ocular phenotype, for which we present data in support of pathogenicity. The m.8340G>A variant occurs within the TψC stem of the mt-tRNA^Lys^ molecule (figure 3D) and as such results in the disruption of Watson-Crick base pairing.^10^ Using a consensus panel of 10 species (figure 3E) to investigate evolutionary conservation, the m.8340G>A variant was shown to affect a highly conserved residue.^11^

A revised version of a previously validated scoring system^12^ was used to assign a pathogenicity classification to this variant. This scoring system uses weighted criteria encompassing biochemical and molecular genetic data to assign a pathogenicity score (out of a total score of 20) to the variant. The m.8340G>A variant is heteroplasmic and has been described previously in one other independent report.^8^ It is shown to affect a highly evolutionarily conserved residue and was absent in maternal tissue samples. Individual enzyme and sequential COX–SDH histochemistry revealed COX-deficient fibres in a skeletal muscle biopsy, with mitochondrial immunofluorescence studies confirming a multiple respiratory chain defect, consistent with a pathogenic mt-tRNA gene variant. Finally, single-fibre analysis demonstrated a statistically significant segregation of mutation load with biochemical deficiency. The m.8340G>A thus scores 15 points with evidence from a gold standard (single-fibre) investigation, and on this basis we would propose that the m.8340G>A is classified as definitely pathogenic.^10 12^

Although the m.8340G>A variant is listed by PhyloTree as occurring in a rare mtDNA haplogroup,^13^ there is no other evidence to support that m.8340G>A may be a neutral polymorphism; this variant is absent from all 32 059 mtDNA GenBank sequences listed on MitoMAP,^3^ while our own diagnostic laboratory (Newcastle Highly Specialised Service for Rare Mitochondrial Disorders) has not detected this variant in over 1200 patients who have undergone whole mitochondrial genome sequencing (unpublished data). We were able to obtain blood, buccal epithelia and urine samples from the patient’s mother all of which failed to demonstrate detectable levels of the m.8340G>A variant. Allowing for the possibility that the mutation may be present in tissues that have not been tested, the absence of the m.8340G>A variant in maternal tissues provides evidence that the mutation is likely to have arisen sporadically. High levels of heteroplasmy within muscle tissue when compared with peripheral tissues, statistically significant segregation of mutation load in single fibres and the absence of the rare mtDNA variant in family members are consistent with this being a pathogenic, de novo variant.

Our case is also associated with an expanded phenotype compared with that described by Jeppesen *et al*,^8^ who previously reported a single patient with the same m.8340G>A variant, but associated with an isolated myopathy. By contrast, the patient whose case we report has multisystemic disease including a history of epilepsy, migraines, growth hormone deficiency, sensorineural deafness and with retinopathy and early-onset bilateral cataracts with biopsy confirming muscle involvement. Of further interest, the patient investigated by Jeppesen and colleagues showed no obvious COX deficiency in muscle, both histochemically and biochemically, with an isolated impairment of respiratory chain complex I. In contrast, our case showed a significant mosaic COX deficiency affecting ~40% of muscle fibres with multiple respiratory chain complex impairment confirmed following immunohistochemistry. This further illustrates the phenotypic variability observed at the level of the biochemical defect, and hence the difficulty in determining a phenotype–genotype correlation, across patients with mtDNA disease.

Retinopathy in the context of mitochondrial disease is well described.^14–17^ Atypical pigmentary retinal dystrophy predominantly affecting the posterior pole is a feature of many mitochondrial diseases (Kearns-Sayre syndrome,^18^ MIDD^19^). Furthermore, fundus autofluorescence imaging showed widespread speckling and areas of atrophy in the posterior pole, which is often described in mitochondrial retinopathies.^20^ The electrophysiology is consistent with abnormalities of the photoreceptors and retinal pigment epithelium.

Disease-causing mtDNA point mutations present a challenge to the clinician both in establishing a diagnosis and further management. Phenotypes are heterogeneous and often overlap. However, patients presenting with both ophthalmic and systemic features should prompt the clinician to consider a mitochondrial aetiology as part of the differential diagnosis. If genetic analysis for common mtDNA mutations does not reveal a mutation, whole mitochondrial genome analysis should be performed with appropriate pretest counselling. Further referrals to relevant specialities may be necessary including cardiology, respiratory, neurology, dietician, nephrology and the diabetes screening service.

In summary, we report a second case of a rare mitochondrial m.8340G>A variant, confirming its pathogenicity on the basis of single-fibre segregation studies and its association with an expanded clinical phenotype. We recommend that the m.8340G>A variant is reclassified as ‘definitely pathogenic’, highlighting the need to carry out further, more extensive testing in patients in whom a mitochondrial diagnosis is suspected. 
